# Harem size should be measured by more than the sum of its parts: Phenology‐based measurements reveal joint effects of intrinsic and extrinsic factors on a polygamous herbivore under non‐stationary climatic conditions

**DOI:** 10.1002/ece3.10865

**Published:** 2024-02-05

**Authors:** Karola Szemán, Zsolt Végvári, Szilvia Gőri, István Kapocsi, Tamás Székely, Jeffrey A. Manning

**Affiliations:** ^1^ Department of Evolutionary Zoology and Human Biology University of Debrecen Debrecen Hungary; ^2^ Centre for Ecological Research Institute of Aquatic Ecology Budapest Hungary; ^3^ Senckenberg Deutsches Entomologisches Institut Muncheberg Germany; ^4^ Hortobágy National Park Directorate Debrecen Hungary; ^5^ Milner Centre of Evolution University of Bath Bath UK; ^6^ School of the Environment, Washington State University Pullman Washington USA

**Keywords:** harem size, phenology, polygynous, Przewalski horse, reserve, wild horse

## Abstract

Social behaviour is thought to be a major component of survival, reproduction, and resilience of populations. Thus, it is a key component in management and conservation of wild populations. In polygynous breeding species, group size influences the reproductive success of males and females, and hence it is essential to understand the environmental and demographic factors that shape the phenology of group size within populations. Here, we investigate harem size and its determinants using a 15‐year dataset of annual harem size phenology‐based metrics from a reintroduced population of wild Przewalski horses in Hortobágy National Park, Hungary. From the initial reintroduction of 21 animals in 1997, the population grew to 174 animals in 2012. During that same period, the number of harems increased from three to 23. Despite the 8‐fold increase in population size, harem sizes remained stable, and variability among harems within years decreased. The annual phenological cycle of harem size was not consistent over the 15‐year period, and the associated annual phenology‐based metrics varied differently over the years. The best predictors of our phenology‐based harem size metrics were adult sex ratio, annual adult mortality and annual mean number of harems, with some evidence that mean age of harem stallions and drought severity were contributing factors. Our findings reveal that complex interactions between demography, climate, and harem size can emerge in social animals. Taken together, our results demonstrate that intrinsic population processes can regulate group size even in the presence of non‐stationary climatic conditions during periods of growth in human‐introduced, semi‐free ranging animal populations.

## Graphical abstract

In our study we investigate harem size phenology and related variation in a large polygamous herbivore, the Przewalski horse, using a 15‐year dataset from an introduced population in Hortobágy National Park, Hungary. Our results demonstrate that intrinsic population processes can play a major role in regulating group size and counter the effects of extrinsic non‐stationary climatic conditions during periods of growth in a group‐living herbivore.

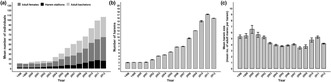

## 1 INTRODUCTION

Harem size represents a major evolutionary component of polygynous mating systems, with foundations in resource selection and female mate choice (Bowyer et al., 2020; Clutton‐Brock, 2016, 2021). Female mate choice in these mating systems forms the basis for female dispersion among mated males, and thus the degree of polygyny (Clutton‐Brock, 2016; Davies et al., 2012), with the benefits of mating with a male being determined by resource availability (i.e. resource defence polygyny) and attributes (Clutton‐Brock, 2016; Davies et al., 2012; Emlen & Oring, 1977; Kerth et al., 2011; Orians, 1969; Verner & Willson, 1966). However, the number of polygynous species that are in decline is on the rise (Braun de Torres et al., 2020; Mittermeier et al., 2007; Rija, 2022; Wakefield et al., 2002), prompting new research into harem size variation and its determinants to inform management, captive breeding and reintroduction efforts worldwide.

Despite past research typically presenting harem size as an annual state variable (e.g. average size of a harem over a given year), the process and timing of harem formation and maintenance are not consistent across polygynous species. Some polygynous species form and maintain harems during the breeding season while others establish and maintain harems year‐round (Clutton‐Brock, 2016). Species that maintain harems year‐round can exhibit varying harem sizes and levels of competition throughout the year (Clutton‐Brock, 2016). Numerous factors may play a role in this variation, including female physiology (e.g. stage of hormonal cycle and breeding status), population demography (e.g. births, deaths, emigration, and immigration), demography of individual harem groups (e.g. births, deaths, new entries, and new departures), and extreme climatic events that influence resources (Aikens et al., 2020; Borkowski, 2000; Buuveibaatar et al., 2013; Forrest & Miller‐Rushing, 2010; Kaczensky et al., 2008; Kaseda & Khalil, 1996; Manning & McLoughlin, 2017; St‐ Louis & Côté, 2017; Sunderesan et al., 2007). Thus, for species that maintain harems year‐round, we view harem size as varying throughout the year, such that a phenological cycle of harem size can emerge for a given population. This view also forms the basis from which researchers can measure various phenology‐based metrics.

Additionally, the magnitude and relative importance of intrinsic and extrinsic factors on harem size may be context specific. For example, non‐stationary climatic conditions may alter the annual availability of resources, which has been shown to alter resource competition, female mate searching, male mating success, and polygyny thresholds in several migratory and non‐migratory polygynous species (Byers et al., 2006; Manning & McLoughlin, 2017). Likewise, factors that determine harem size in free‐ranging species that undergo long‐distance migrations to track seasonal resources (Dingle, 2014) may differ from those that underlie harem size in similar species restricted to local climate conditions when introduced into fenced conservation reserves (e.g. American plains bison (*Bison bison bison*) introduced to Badlands National Park, South Dakota; Dingle, 2014). Despite this awareness, our understanding of harem size variation in polygynous migratory populations confined to reserves is hindered by a lack of empirical studies that explicitly link harem size metrics derived from its annual phenological cycle to local demography and meteorological factors.

Building on our view of a within‐year phenological cycle of harem sizes and upon the insights of Verner and Willson (1966), Orians (1969), Emlen and Oring (1977), Clutton‐Brock (2016), and Manning and McLoughlin (2017), we investigated the relative importance of demographic and meteorological determinants on four separate annual phenology‐based measures of harem size from a 15‐year study of a reintroduced population of Przewalski horses (*Equus ferus przewalskii*) in Pentezug Reserve, Hortobágy National Park (HNP), Hungary. The Przewalski horse is a true wild horse that was discovered at the end of the 19th century and became extinct in the wild 70 years later. Several individuals subsequently discovered in zoos were used for captive breeding and reintroduction into the wild. They exhibit year‐round female‐defence polygyny characterised by relatively stable, non‐territorial harem groups consisting of a dominant male (harem stallion) that maintains a harem of females and their offspring (Bouman, 1986; King, 2002). This social system is similar to some primates (Parnell, 2002; Smuts & Smuts, 1993), feral horses in North America and Australia (Manning et al., 2015; Ransom & Kaczensky, 2016; Scorolli & Lopez Cazorla, 2010), American bison (Wolff, 1998), and other ungulates (Bowyer et al., 2020; Clutton‐Brock, 2016, 2021). They are sexually monomorphic, with both sexes selecting mates and males not possessing ornaments, indicating that females choose mates according to resource quality associated with each male. Recent evidence from feral horses (Contasti et al., 2013; Manning & McLoughlin, 2019) indicates that peaks, lulls, and stable periods in harem size may occur within the annual harem cycle of Przewalski horses, which may also be the case with other polygynous species (e.g. non‐human primates (Smuts & Smuts, 1993) and other ungulates (Bonenfant et al., 2004; Kaseda & Khalil, 1996)).

We developed four harem size metrics, where each represented a unique attribute of the 12‐month phenological cycle of harem size (Table 1). We used linear modelling in a parallel analysis framework to test multiple working hypotheses regarding the effects of meteorological and demographic conditions on each harem size phenology metric (Table 2). We predicted that average harem size over each 12‐month period (which represents the commonly viewed steady state of harem size) would be positively related to overall population size more so than other demographic covariates or meteorological conditions because increased population size coincides with larger numbers of females being available to join harems. Annual maximum harem size (the largest monthly mean harem size within a 12‐month cycle) is expected to be negatively related to the number of adult stallions in the population due to higher levels of male–male competition. As the number of male competitors increases, it becomes increasingly difficult for males to increase harem size (Manning & McLoughlin, 2019). Additionally, if the number of potential harem stallions is higher in the population, females may choose solitary stallions or smaller harems to avoid within‐group competition. Further, we expected that within‐year dispersion (variability) of harem sizes among stallions would increase with increased drought severity, as water availability can accentuate male mating inequality and opportunity for sexual selection (Manning & McLoughlin, 2017, 2019).

**TABLE 1:** Definition and justification of harem size variables (dependent variables) used in the study.

Dependent variable	Definition	Hypothesis
Annual mean harem size	The mean number of adult females per harem group during the year, calculated from data from monthly surveys	In a larger population predict a larger pool of females who potentially join a harem group, therefore annual mean population size would positively react to increasing population size
Maximum harem size	Largest monthly mean during a 12‐month period	A larger number of adult stallions in the population could lead to a higher level of intrasexual competition which makes it more challenging to tend big harem group for a long time and the maximum harem size would be smaller. Alternatively, with a higher number of potential harem stallions in the population females may choose solitary stallions or smaller harems to avoid intragroup competition
Dispersion (CV) of harem sizes	Within‐year dispersion of harem sizes, calculated as the coefficient of variation in mean monthly harem size each year (${\mathrm{CV}}_{\text{mmhs}}=\left(\mathrm{SD}/\bar{x}\;\text{monthly harem size}\right)\times 100$)	Within‐year dispersion (variability) of harem sizes and HSMDI are expected to increase with increased drought severity, as water availability can accentuate male mating inequality and opportunity for sexual selection
Harem size monthly departure index (HSMDI)	Difference in annual mean harem size from the long‐term (15‐year) mean harem size ( 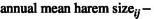 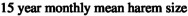 )	

**TABLE 2:** Description of explanatory variables.

Explanatory variables	Definition
Adult sex ratio (ASR)	The proportion of adult males in the population: (males ≥3 years old/[males ≥3 years old + females ≥2 years old])
Annual adult mortality (Mort_a,a_)	The number of adult horse mortalities reported to HNP personnel within a 12‐mo period/mean annual size of the adult population
Annual juvenile female mortality (Mort_a,jf_)	Dividing the number of juvenile female mortalities reported to HNP personnel by the annual mean number of juvenile females in the entire population
Annual mean age of harem stallions (MA_a,hs_)	The mean age of harem holding stallions during each 12‐month period
Annual mean number of harems (NrH_a_)	Annual mean number of harems from monthly counts conducted each year
Drought severity index (DSI)	We standardised average temperature and total precipitation and calculated the standardised average temperature – standardised total precipitation, such that higher (positive) values corresponded to relatively hotter and drier drought conditions, whereas lower (negative values) characterised relatively cooler and wetter conditions (Figure 2). We then summed the number of months with values >0 within a year to represent the number of months above normal drought conditions each year and used this as a DSI in our models

## 2 MATERIALS AND METHODS

### 2.1 Study system and organism

We studied a naturally regulated population of Przewalski horses within the 2450‐ha Pentezug Reserve, a grassland steppe system within Hortobágy National Park (HNP) in eastern Hungary (47°51′75 N, 21°09′28 E). The Pentezug Reserve is characterised by relatively flat terrain (about 87 m ASL) supporting native, alkali steppe vegetation communities, including grasslands, meadows, marshes and forests (Deák, Valkó, Alexander, et al., 2014; Deák, Valkó, Török, & Tóthmérész, 2014; Deák, Valkó, Tóthmérész, & Török, 2014; Zimmermann et al., 1998). The climate is continental, with four distinct seasons and average temperatures ranging from −2.5°C (min. −28°C) in January to 21°C (max. 38°C) in July, average rainfall of 50, and 2–10 cm of winter snow (average of 40–45 days per year). Two small, perennial rivers provide year‐round drinking water for horses, with autumn and spring rains providing temporary ponds (Zimmermann et al., 1998). Climatic conditions are becoming increasingly non‐stationary (e.g. decreasing level of summer rains, increasing temperature), with evidence over the past decade of effects on phenology of wildlife migration (Végvári & Kovács, 2010).

The reserve is bounded by a 3‐wire, New Zealand style electric fence (24.8 km long, 1.4 m high; Gallagher, Budapest, Hungary), which restricts large mammal immigration and emigration while allowing smaller wildlife (e.g. roe deer (*Capreolus capreolus*), wild boar (*Sus scrofa*), red fox (*Vulpes vulpes*) and badger (*Meles meles*)) to move unimpeded. In addition to Przewalski horses, Heck cattle (*Bos primigenius taurus*) are free ranging within the reserve (Zimmermann et al., 1998, 2009). The horses and cattle graze year‐round throughout the reserve without supplemental forage or veterinary care (since 2001) (Kerekes et al., 2021).

Originally introduced in 1997 as part of an international breeding program (Kerekes et al., 2019), Pentezug's Przewalski horse population is without harvest, round ups, or removal by humans and free of natural predation. There are no large carnivores (felids or canids) in the reserve. The founder population of horses introduced to Pentezug Reserve consisted of 21 horses (13 mares and 8 stallions), which were introduced from different European zoos over a 5‐year period, beginning in 1997 (Kerekes et al., 2019). To increase genetic diversity, one additional mare was translocated to the reserve in 2007 and one stallion in 2010.

Changes in climate can influence resource availability, body condition, and mortality of large, harem‐forming herbivores in this system (Kerekes et al., 2019), with implications on the acquisition, composition, and size of Przewalski horse harems, as also seen in feral horses (Manning et al., 2015; Manning & McLoughlin, 2017). In addition to these external forces, intrinsic population processes likely also determine harem size within and among years, similarly to those expected in other polygynous species translocated to conservation reserves (Beck, 2016; Dingle, 2014). Intrinsic processes that underlie the 12‐month‐cycle of harem phenology in Przewalski horses in the reserve include the dispersal of 18–24‐month‐old female offspring from their natal harem to another harem, the expulsion of subadult males from their natal harem, adult mortality, and displacement of a dominant stallion by another from outside the harem (Bouman, 1986). Female offspring may also leave their natal harem to join a bachelor stallion or other harem for an extended period and later return to their natal harem, and this can happen multiple times before the female settles (Bouman, 1986). These likely have additive and interactive effects with extrinsic processes that shape the 12‐month phenological cycle of Przewalski horse harem sizes that evolutionarily evolved through ecological relationships between the distribution of resources, habitat, and feeding style (Szemán et al., 2021).

Initially after reintroduction, the horses formed spatially distinct harems of one to eleven mares led by individual stallions (Kerekes et al., 2019). Stallions without harems live solitarily or form loose, ephemeral bachelor bands (Bouman, 1986). Since 2001, the population experienced positive population growth due to reproduction; immunocontraception of mares began in 2013 (Kerekes et al., 2021). Subsequently, the population exhibited behavioural synchrony, such that animals were coordinating with conspecifics within and among harems (Kerekes et al., 2021; Maeda et al., 2021). The relatively distinct harem and bachelor groups that were present during our study period provided a unique opportunity to investigate the singular and additive effects of intrinsic and extrinsic factors that influence the phenology of harem size during a period of positive population growth after initial introduction and prior to behavioural synchrony.

### 2.2 Field data collection

We used an individual‐based, longitudinal dataset of monthly field observations of the entire population of Pentezug's Przewalski horses from May 1998 to December 2012, obtained from the HNP Directorate. Park personnel have used standardised, non‐invasive sampling protocols and a photographic database to monitor horses since their introduction in 1997. Over the study period, age, sex, breeding status, and harem affiliation were recorded from 267 individuals (39 harems comprised of 174 adults and 93 subadults). Harems and individual horses were observed with binoculars from a vehicle or on foot from 5 to 30 m at least once a month (for details please see Kerekes et al., 2021). Horses were individually identified according to phenotypic traits (i.e. sex, colour, stripes, whorls, mane position (left or right), belly colouration and specific marks such as scarfs, white marks or special colour patterns; Kerekes et al., 2019, 2021; Zimmermann et al., 2009). Mares were assigned to harems based on their social interactions (i.e. driving, following, and mutual grooming; Glatthaar, 2012) with other mares in a harem or with a dominant stallion. During each observation, individual IDs of all harem members were recorded.

As young stallions and mares disperse from their natal harem before they reproduce, and the age at first reproduction in mares in this population was two years, we defined an adult female as being ≥2 years old and not being affiliated with its natal harem. We considered stallions that were ≥3 years old and had left their natal harem to have entered the adult stallion age class. As our focus was on the composition of breeding individuals within and among harems, we classified sub‐adults each year as those that had not left their natal harem before they had reached these age classes and excluded them from analyses.

### 2.3 Harem phenology

**FIGURE 1:**
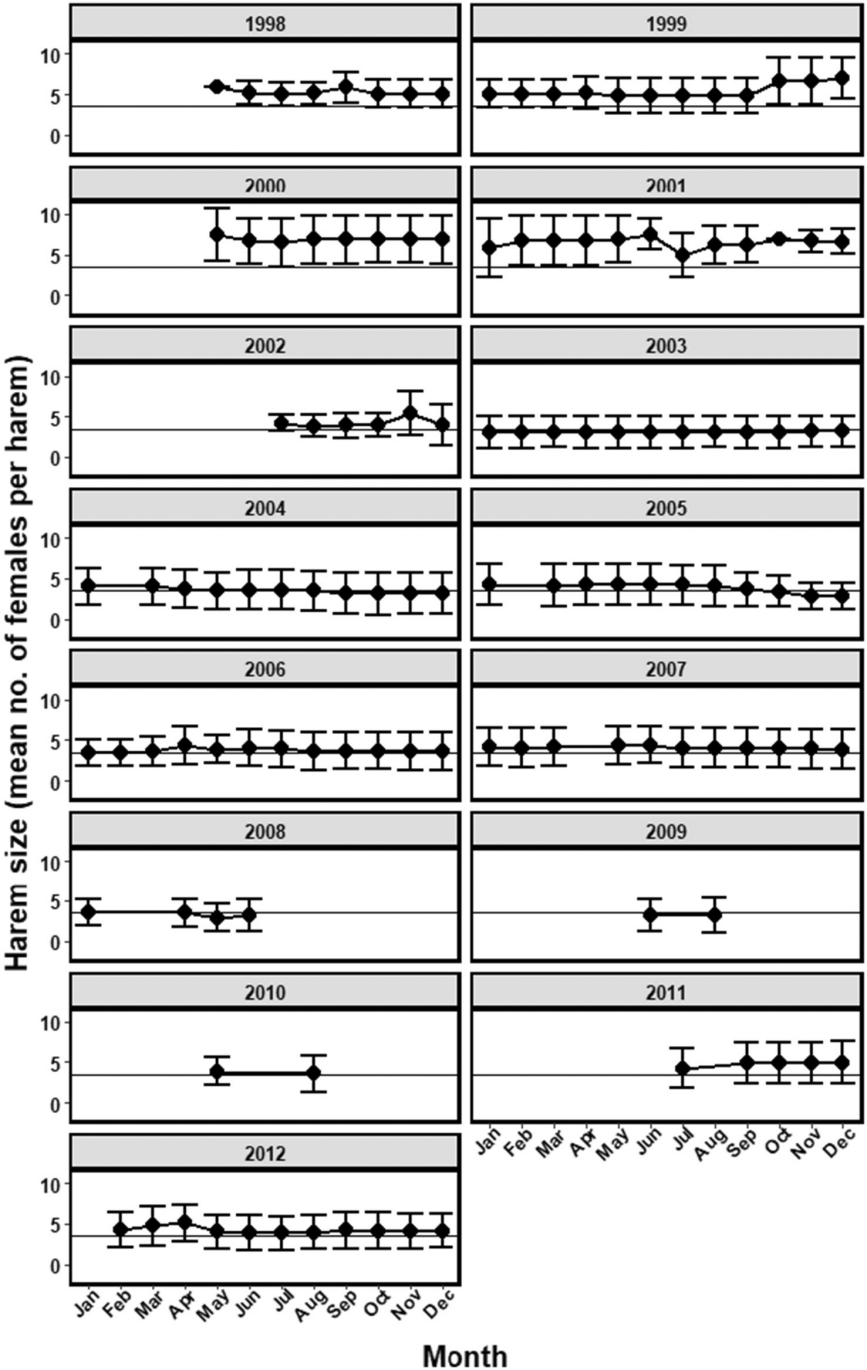
Harem sizes in Przewalski horses in Pentezug Biosphere Reserve, Hortobágy National Park, Hungary between 1998 and 2012. The horizontal line represents the overall mean harem size over the 13 years (3.46 mares per harem).

We used mean harem size each month to generate a 12‐month phenology of harem sizes each year (Figure 1), from which we calculated annual mean harem size and three phenology‐based harem size metrics. Annual mean harem size represented the average, commonly viewed steady state of harem size over a 12‐month period, which enabled us to evaluate how intrinsic and extrinsic factors influenced inter‐annual patterns in the within‐year steady state of harem size. We also calculated the annual maximum harem size, defined as the largest monthly mean harem size within a 12‐month cycle. We assumed that years with a relatively high maximum harem size reflected relatively better environmental and meteorological conditions that enabled stallions to retain and increase harem sizes and greater male mating success (Manning & McLoughlin, 2017). Additionally, we calculated the within‐year dispersion of harem sizes as the coefficient of variation in mean monthly harem size each year (${\mathrm{CV}}_{\text{mmhs}}=\left(\mathrm{SD}/\bar{x}\;\text{monthly harem size}\right)\times 100$) (Figure S1). We assumed that years with relatively greater dispersion reflected an increased degree of male mating inequality and opportunity for sexual selection (Emlen & Oring, 1977), which has been shown to emerge in feral horses from factors including local demography and drought conditions (Manning & McLoughlin, 2017, 2019). Lastly, because harem size is expected to vary within and among years, we calculated a monthly harem size metric for each year by first measuring the difference in mean monthly harem size (the *i*th month of the *j*th year) from the long‐term average monthly harem size over the 15‐year period (mean harem_*ij*_ – 15 year monthly mean harem). We then summed the number of months in each year that were below the 15‐year average, and considered this as a harem size monthly departure index (HSMDI). An HSMDI = 0 indicated a year when all monthly average harem sizes were larger than the long‐term normal harem size (Figure 1). We summarise our harem phenology metrics in Table 1. Due to low sample size in 2009 and 2010 (*n* = 2 observations each year), these two years were excluded from analyses.

### 2.4 Explanatory variables

To investigate how factors intrinsic to the population may influence the four phenology‐based harem size metrics, we calculated empirical estimates of five demographic parameters annually. As harem size and the probability of acquiring harems have been linked to the adult sex ratio (ASR; proportion of adult males in the population) in other large ungulates (Bonenfant et al., 2004; Kaseda & Khalil, 1996; Manning & McLoughlin, 2019), we calculated ASR annually as the 12‐month mean of the monthly proportion of adult stallions in the entire population (males ≥3 years old/[males ≥3 years old + females ≥2 years old]) (Ancona et al., 2017; Andersson, 2004; McNamara et al., 2000). We calculated annual adult mortality as the number of adult horse mortalities reported to HNP personnel within a 12‐month period/mean annual size of the adult population. The mortality data were recorded during weekly visual monitoring of the horses by HNP personnel within the fenced park, where horses were identified at the individual level within groups throughout the study period. This involved targeted searches for every individual horse not detected during a weekly survey until either the animal was located alive or dead or several months had past, at which time the missing individual would be recorded as dead. The open grassland and gentle topography minimised missing animals and bias in our estimates of mortality. We also used this data to calculate annual juvenile female mortality. For this, we calculated a juvenile female mortality rate per‐capita by dividing the number of juvenile female mortalities reported to HNP personnel by annual mean number of juvenile females in the entire population. In the analyses, we used this predictor with a 1‐year (*t −* 1) time lag to consider the reduction in new adult females available for joining harems the following year. Because age can influence a stallion's ability to retain harems and mares within harems (Bouman, 1986), we calculated the mean age of harem holding stallions during each 12‐month period. We used this variable as a second‐degree polynomial function in our analyses to reflect a gradual threshold in harem acquisition and retention due to stallion senescence, creating a ∩‐shaped correlation between age, resource holding abilities, and paternity success (Mainguy & Côtè, 2008; Mysterud et al., 2003; Perlman et al., 2016; Setchell et al., 2006; Silk et al., 2020; Van Noordwijk & Van Schaik, 1985; Watts, 2019; Yoccoz et al., 2002). Lastly, we calculated the annual mean number of harems from monthly counts conducted each year.

**FIGURE 2:**
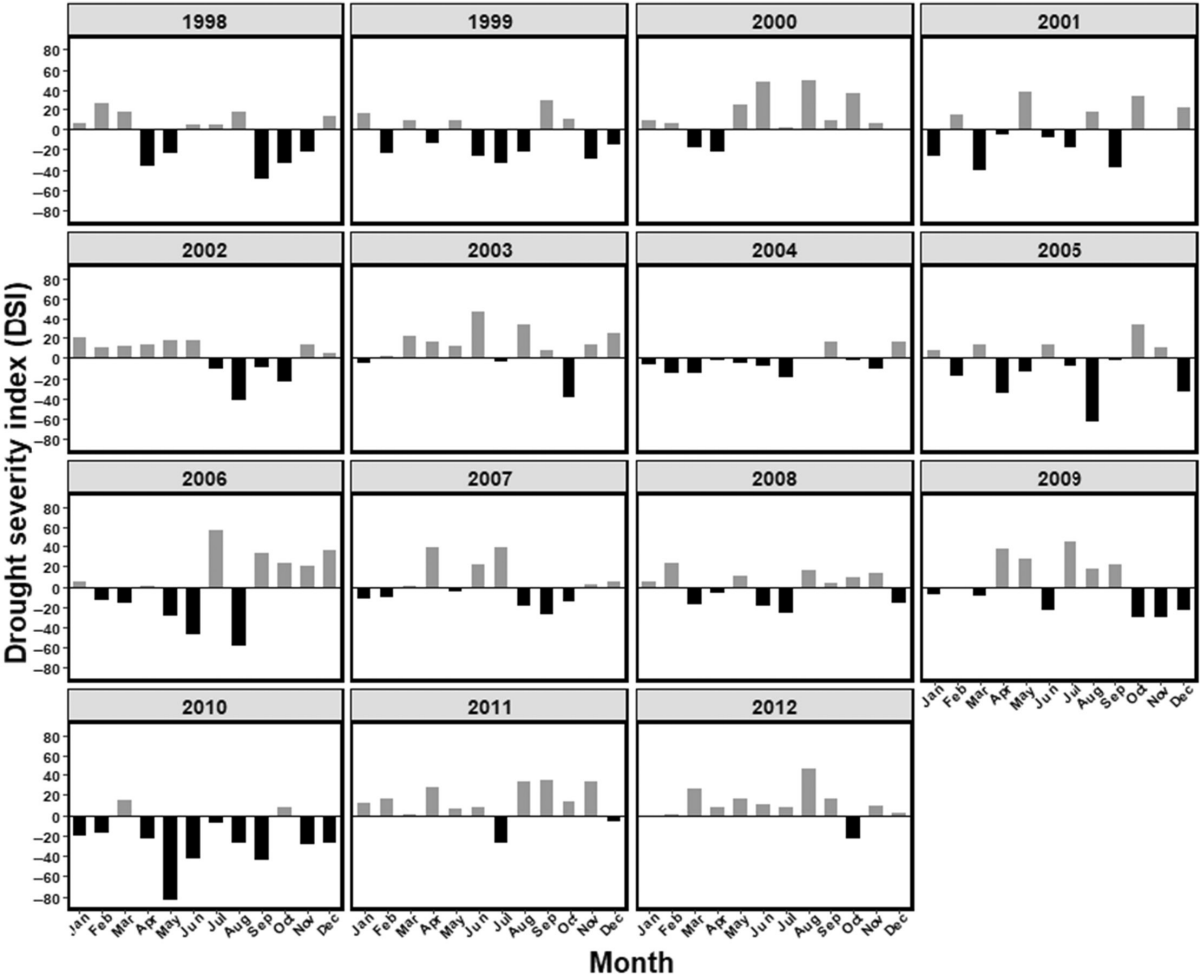
Drought severity index (DSI) in the study area between 1998 and 2012. Grey colour represents the years with positive DSI values while black colour represents the years with negative DSI values.

Because climatic conditions have been found to underly ASR and polygyny thresholds in feral horses (Manning et al., 2015; Manning & McLoughlin, 2017), we also developed an annual drought severity index (DSI) from measures of average monthly temperature and total precipitation obtained from worldclim.org. Here, DSI served as an index of variable food quality and subsequent body condition over the year, which we posited may influence female mate choice, and hence harem size and retention by stallions. We reasoned that the most severe drought conditions would be those with hottest temperatures and lowest total precipitation. We used the climate rasters with resolutions of 0.5° × 0.5°, retrieved from worldclim.org and selected the single grid covering the whole of the study area. In the following step we extracted the climate values from this raster grid. For each month, we standardised average temperature and total precipitation, as monthly mean temperature or total precipitation—15‐year average temperature or precipitation. Then, we calculated the standardised average temperature – standardised total precipitation, such that higher (positive) values corresponded to relatively hotter and drier drought conditions, whereas lower (negative values) characterised relatively cooler and wetter conditions (Figure 2). We then summed the number of months with values >0 within a year to represent the number of months above normal drought conditions each year and used this as a DSI in our models (see Table 2 for details).

### 2.5 Statistical analysis

We developed four parallel analyses to evaluate the importance of demographic and meteorological factors on variation in harem size. For each phenology‐based harem size metric, we constructed an a priori candidate set of 16 linear models based on biologically relevant combinations of intrinsic and extrinsic covariates (Anderson & Burnham, 2002). For HSMDI, we used a negative binomial model; the other three response variables were modelled using ordinary least squares regression. We tested for bivariate correlations among the covariates and found all Spearman's |*r*| values to be <.7 (Table S1). We considered 0.7 as a threshold for collinearity because collinearity above this value begins to severely distort model estimation and subsequent prediction in ecological studies (Dormann et al., 2013; Tabachnick et al., 2007). While there are many options on how to statically handle this situation (Gregorich et al., 2021), given these results and our small sample sizes, we constrained the model sets to 2‐structural parameter additive models. We used Information Theory with the second‐order small‐sample bias‐corrected Akaike's Information Criterion (AICc) and AICc weights ($w_{\text{AICc}}$) (Anderson & Burnham, 2002) to quantify the support of each model within each parallel analysis using the bbmle package in R (version 4.1.2; Bolker, 2022; R Core Team, 2021). We tested for normality of residuals (marginal residuals vs. fitted values and Q–Q plots) in the most supported model in each parallel analysis and also assessed the *r*‐square, *F*‐statistic, and *p*‐value metrics of these models. Following Akaike (1973) and Anderson and Burnham (2002), we considered the model with the lowest ΔAICc‐value and highest $w_{\text{AICc}}$ within a candidate set as having the greatest empirical support for inference regarding the coinciding phenology‐based measure of harem size. We considered models with ΔAICc ≤2 as competing.

## 3 RESULTS

**FIGURE 3:**
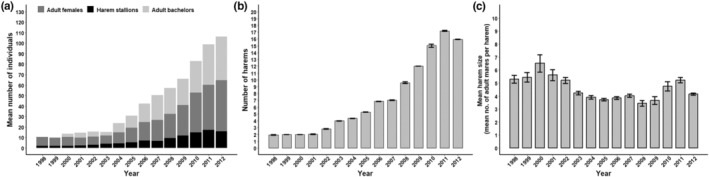
Changes in the number of horses (a), number of harems (b) and mean harem size (c) of Przewalski horses between 1998 and 2012. 95% confidence intervals are represented by black lines.

The initial population size of 21 adults reintroduced in 1998 successfully reproduced and thus the population included 174 adults by 2012 (Figure 3a). As the population increased, the number of harems also increased from the original 3 in 1998 to 23 in 2012 (Figure 3b), producing a total of 39 harems over the entire study period. Despite the increased population and number of harems, harem size did not increase over time. Instead, mean harem size each year ranged by only a few adult mares (Figure 3c and Figure S1), indicating a temporal decoupling of population size and mean harem size as the population grew. Harem sizes ranged from 1 to 11 mares (95% confidence interval [4.192, 4.109]), monthly mean harem sizes ranged from 2 to 7 mares (95% confidence interval [4.761, 4.385]), and different peaks and periods of stability emerged within the 12‐month (January 1–December 31) phenological harem cycle and relative to the long‐term average (i.e. normal) harem size (Figure S1). Approximately half of the adult males held a harem in at least one time period during their lifetime (*n* = 39 stallions), and the remaining adult males remained as bachelors (Figure 3a).

### 3.1 Factors influencing annual average harem size

**FIGURE 4:**
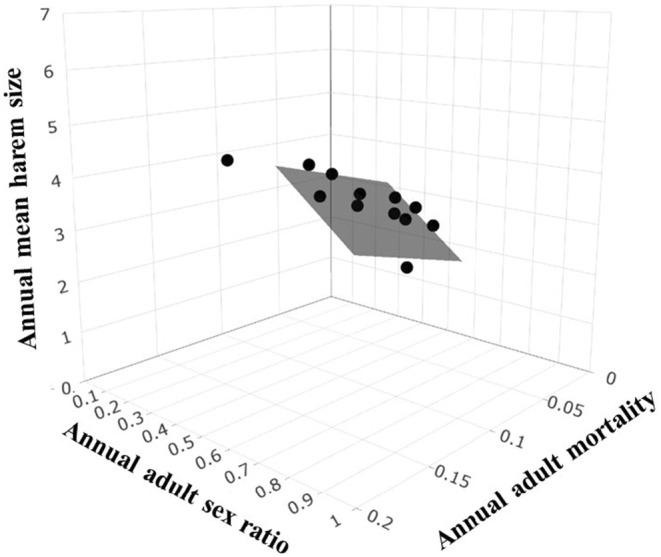
Annual mean harem size of Przewalski horses predicted by the additive effects of adult sex ratio and annual adult mortality between 1998 and 2012 (ordinary least squares regression model *w*_AICc_ = 0.98).

Annual mean harem size was best predicted by the additive effects of ASR and annual adult mortality (annual mean harem size model *w*_AICc_ = 0.98; Table 3); this model fit the data well and exhibited a symmetric pattern of marginal residuals versus fitted values (i.e. normality; Figure S2). For every 10% increase in ASR, mean harem size decreased by 0.479, and every 10% increase in adult mortality led to mean harem size increasing by 0.877 ($\text{mean harem size}=4.28-4.79\left(\mathrm{ASR}\right)+8.77\left(\text{adult mortality}\right)$; adjusted *R* ^2^ = .91, *F*_2,9_ = 59.62, *p* < .001; Figure 4). There was no evidence that other demographic processes or extrinsic factors influenced mean harem size (all ΔAICc ≥2; Table 3).

**TABLE 3:** Parallel analyses of phenology‐based harem size metrics, including linear regression models, explanatory variables, and second‐order Akaike information criterion statistics.

Phenology‐based harem size model	Explanatory variables	*k* ^a^	Log‐ likelihood	ΔAICc^b^	$w_{\text{AICc}}$ ^c^
Annual mean harem size	**ASR + Mort**_**a,a**_	**3**	**4.29**	**0.00**	**0.98**
	ASR + MA_a,hs_	3	0.14	8.31	0.02
	ASR + MA_a,hs_ + (MA_a,hs_)^2^	4	1.47	11.93	0.00
	ASR	2	−4.61	13.09	0.00
	MA_a,hs_ + (MA_a,hs_)^2^	3	−3.93	16.44	0.00
	Mort_a,a_	2	−6.44	16.76	0.00
	DSI + ASR	3	−4.13	16.85	0.00
	MA_a,hs_	2	−6.59	17.05	0.00
	ASR + Mort_a,jf_	3	−4.56	17.71	0.00
	ASR + NrH_a_	3	−4.59	17.77	0.00
	DSI + MA_a,hs_	3	−6.27	21.13	0.00
	DSI + MA_a,hs_ + (MA_a,hs_)^2^	4	−3.91	22.70	0.00
	Mort_a,jf_	2	−10.94	25.74	0.00
	DSI	2	−11.52	26.91	0.00
	NrH_a_	2	−11.59	27.04	0.00
	DSI + NrH_a_	3	−11.23	31.05	0.00
Maximum harem size	**ASR + NrH**_**a**_	**3**	**−10.32**	**0.00**	**0.37**
	**ASR**	**2**	**−12.96**	**0.57**	**0.28**
	**DSI + ASR**	**3**	**−11.22**	**1.81**	**0.15**
	ASR + Mort_a,a_	3	−11.81	2.98	0.08
	ASR + MA_a,hs_	3	−12.33	4.03	0.05
	ASR + Mort_a,jf_	3	−12.49	4.35	0.04
	MA_a,hs_	2	−17.01	8.68	0.00
	Mort_a,a_	2	−17.15	8.96	0.00
	ASR + MA_a,hs_ + (MA_a,hs_)^2^	4	−12.32	10.29	0.00
	Mort_a,jf_	2	−17.88	10.41	0.00
	MA_a,hs_ + (MA_a,hs_)^2^	3	−16.17	11.71	0.00
	DSI + MA_a,hs_	3	−16.22	11.81	0.00
	DSI	2	−18.58	11.82	0.00
	NrH_a_	2	−18.61	11.87	0.00
	DSI + NrH_a_	3	−18.43	16.23	0.00
	DSI + MA_a,hs_ + (MA_a,hs_)^2^	4	−15.73	17.11	0.00
Dispersion (CV) of harem sizes	**NrH**_**a**_	**2**	**−43.13**	**0.00**	**0.51**
	Mort_a,jf_	2	−44.91	3.54	0.09
	ASR + NrH_a_	3	−42.66	3.76	0.08
	DSI	2	−45.03	3.80	0.08
	ASR	2	−45.34	4.41	0.06
	Mort_a,a_	2	−45.37	4.47	0.05
	DSI + NrH_a_	3	−43.12	4.69	0.05
	MA_a,hs_	2	−45.50	4.73	0.05
	DSI + ASR	3	−44.82	8.09	0.01
	ASR + Mort_a,jf_	3	−44.87	8.19	0.01
	ASR + Mort_a,a_	3	−44.96	8.37	0.01
	DSI + MA_a,hs_	3	−45.00	8.46	0.01
	ASR + MA_a,hs_	3	−45.16	8.77	0.01
	MA_a,hs_ + (MA_a,hs_)^2^	3	−45.17	8.78	0.01
	ASR + MA_a,hs_ + (MA_a,hs_)^2^	4	−44.17	13.07	0.00
	DSI + MA_a,hs_ + (MA_a,hs_)^2^	4	−44.20	13.12	0.00
Harem size monthly departure index (HSMDI)	**ASR**	**2**	**−18.05**	**0.00**	**0.44**
	ASR + MA_a,hs_ + (MA_a,hs_)^2^	4	−13.92	2.74	0.11
	ASR + Mort_a,a_	3	−17.27	3.15	0.09
	ASR + NrH_a_	3	−17.28	3.16	0.09
	ASR + MA_a,hs_	3	−17.32	3.25	0.09
	DSI + ASR	3	−17.45	3.51	0.08
	ASR + Mort_a,j_	3	−17.67	3.94	0.06
	Mort_a,a_	2	−20.78	5.45	0.03
	MA_a,hs_	2	−21.77	7.44	0.01
	MA_a,hs_ + (MA_a,hs_)^2^	3	−21.52	11.65	0.00
	NrH_a_	2	−23.92	11.74	0.00
	DSI + MA_a,hs_	3	−21.67	11.94	0.00
	DSI	2	−24.33	12.56	0.00
	Mort_a,jf_	2	−24.39	12.68	0.00
	DSI + NrH_a_	3	−23.85	16.30	0.00
	DSI + MA_a,hs_ + (MA_a,hs_)^2^	4	−21.38	17.66	0.00

*Note*: Bold text emphasises the model with the greatest support (e.g. likelihood and AICc weight) for inference. Explanatory variables were annual mean number of harems (NrH_a_), annual adult mortality (Mort_a,a_), annual juvenile female mortality (Mort_a,jf_), mean age of harem stallions (MA_a,hs_), adult sex ratio (ASR; proportion of adult males in the population) and a drought severity index (DSI).

^a^ Number of structural parameters (predictor variables plus intercept).

^b^ Difference in second‐order Akaike's information criterion (ΔAICc) from the most parsimonious model.

^c^ Small sample bias corrected second‐order Akaike's information criterion (AICc) weight.

### 3.2 Factors influencing annual maximum harem size

**FIGURE 5:**
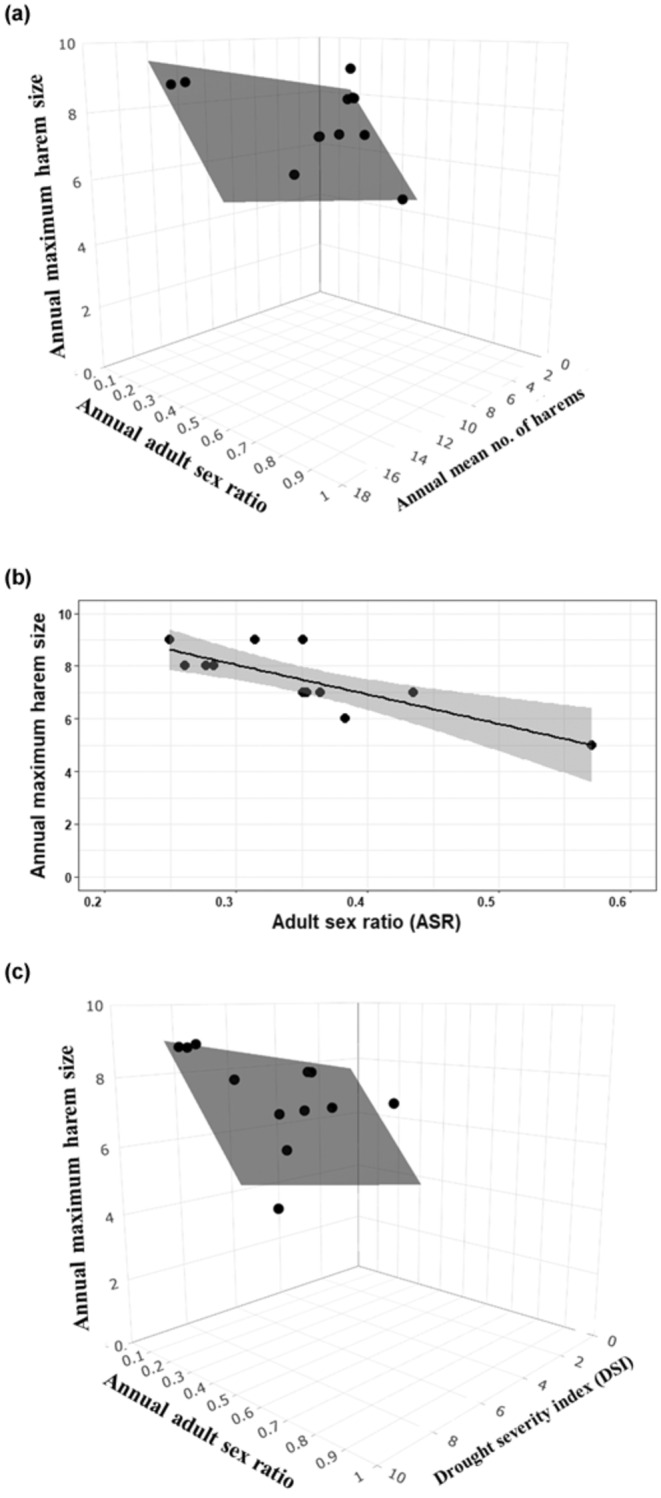
Graphical representations of 3 competing ordinary least squares regression models predicting the effects of intrinsic and extrinsic factors on annual maximum harem size of Przewalski horses between 1998 and 2012. (a) model adult sex ratio + annual mean number of harems (ΔAICc = 0.00), (b) model adult sex ratio (ΔAICc = 0.57), and (c) model adult sex ratio + drought severity index (ΔAICc = 1.81); (Σ*w*_AICc_ = 0.80).

Three competing models explained annual maximum harem size (model ASR + annual mean number of harems (*w*_AICc_ = 0.37), model ASR (ΔAICc = 0.57, *w*_AICc_ = 0.28), and model DSI + ASR (ΔAICc = 1.81, *w*_AICc_ = 0.15); Table 3). These three models contained ASR and had a combined *w*_AICc_ = 0.80, providing strong evidence that ASR is an important predictor of annual maximum harem size. The presence of annual mean number of harems and DSI in the more complex competing models indicate that these intrinsic and extrinsic forces may also have underlying effects on annual maximum harem size. However, because these models were nested, we considered the simpler model (model ASR) as the most parsimonious for making inference. This model fit the data well (i.e. normality; Figure S3) and predicted that each 10% increase in ASR led to a decrease in maximum harem size of 1.24 females ($\text{maximum harem}=11.43-11.24\left(\mathrm{ASR}\right)$; adjusted *R* ^2^ = .61, *F*_1,10_ = 17.92, *p* = .002; Figure 5). There was little evidence that other demographic factors influenced annual maximum harem size (all other models ΔAICc ≥2; Table 3).

### 3.3 Factors affecting variation in harem size (CV)

**FIGURE 6:**
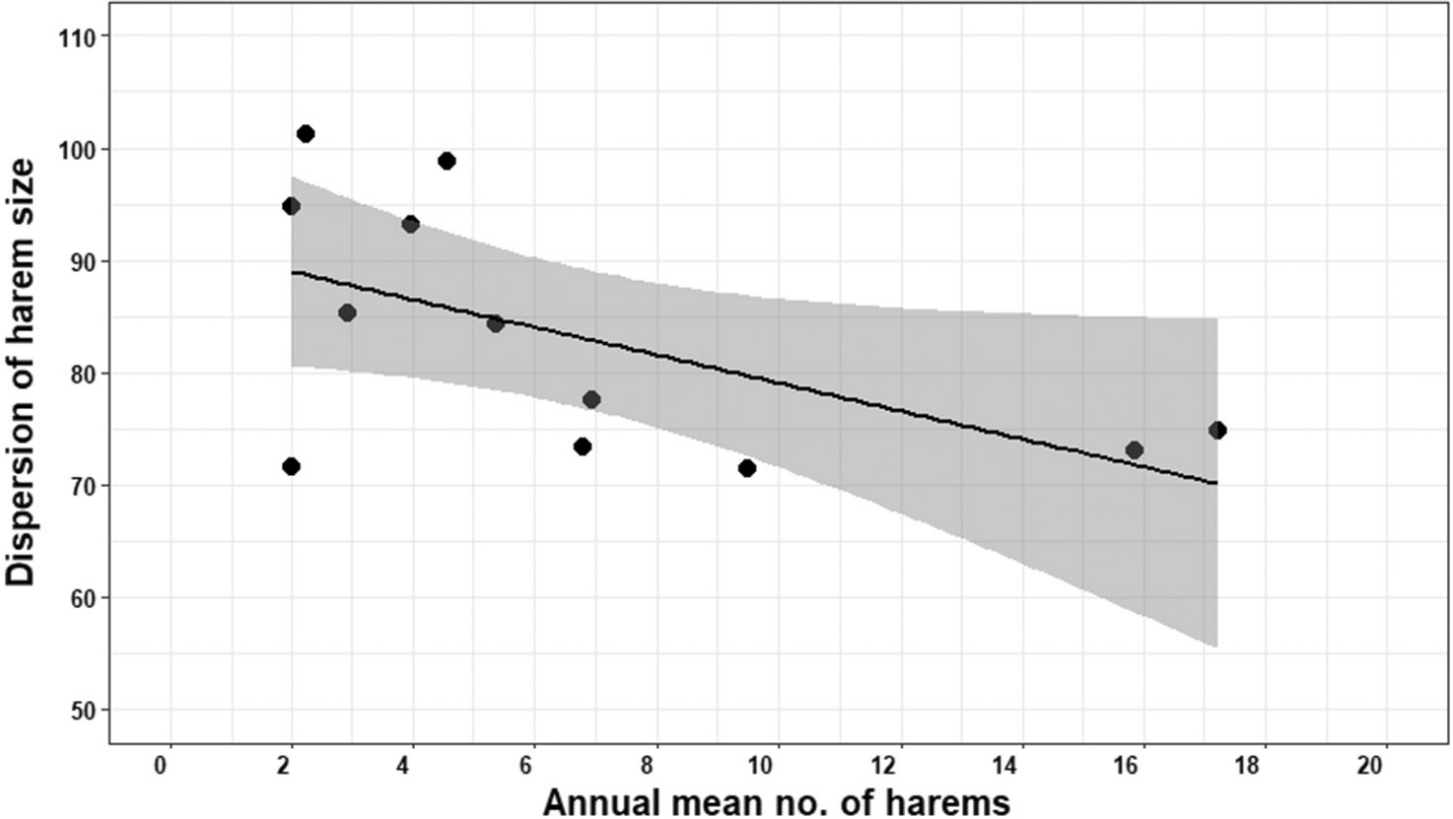
Variance of harem size in relation to annual mean number of harems between 1998 and 2012 (ordinary least squares regression model *w*_AICc_ = 0.51).

Annual variation in dispersion of harem sizes was explained best by annual mean number of harems (*w*_AICc_ = 0.51; Table 3), which fit the data well (Figure S4). We found no evidence that extrinsic or other intrinsic factors affected the dispersion of harem sizes (all ΔAICc ≥2; Table 3). This ‘best’ supported model predicted that for every 1 unit increase in annual mean number of harems, the dispersion of harem sizes decreased by 1.24% during the study period ($\text{variation of harem sizes}=91.50-1.24\left(\text{annual mean number of harems}\right)$). However, this model explained a low amount of variation in annual dispersion of harem size (adjusted *R* ^2^ = .26, *F*_1,10_ = 4.90, *p* = .05; Figure 6).

### 3.4 Factors affecting annual deviations from the long‐term, normal harem size (HSMDI)

**FIGURE 7:**
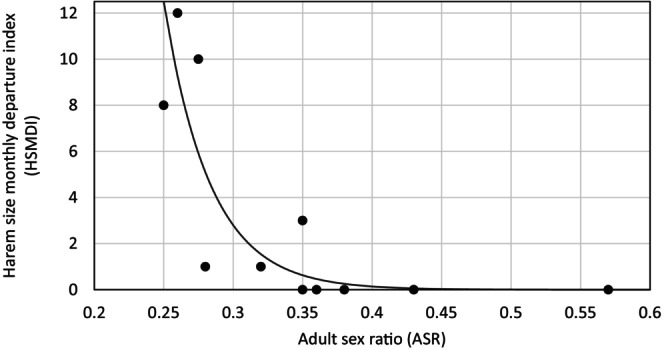
Harem size monthly departure index as a function of adult sex ratio between 1998 and 2012 (generalised linear negative binomial model *w*_AICc_ = 0.44).

Our most supported negative binomial model of harem size monthly departure index contained the single effect of ASR (*w*_AICc_ = 0.44; Table 3, Figure S5) ($\text{HSMDI}=10.01-29.98\left(\mathrm{ASR}\right)$; Figure 7). In addition to ASR, there was some slight evidence of an additive quadratic effect of mean age of harem stallions on HSMDI (ΔAICc = 2.74; *w*_AICc_ = 0.11; Table 3). There was little evidence of other demographic processes or extrinsic factors influencing HSMDI (Table 3).

## 4 DISCUSSION

Harem size is an important evolutionary component of the polygynous mating system, but akin to population size, it also exhibits within‐year variation due to turnover in individuals through time (Clutton‐Brock, 2016), and can be viewed as a demographic parameter. Viewing harem size from these perspectives, we used a 15‐year dataset from a wide‐ranging, polygynous herbivore introduced to a large, Hungarian steppe wildlife reserve to calculate four harem size metrics. Each metric represented a different measure of the 12‐month phenological harem size cycle and show that multiple demographic factors emerge as the primary determinants of harem size, sometimes with opposing or accentuating effects. Specifically, skewed ASRs toward males reduced annual mean harem size, annual maximum harem size, and HSMDI. We also found that annual adult mortality increased annual mean harem size, thus opposing the negative effects of ASR. There was also evidence that annual mean number of harems increased maximum harem size and decreased annual variation in harem sizes. Additionally, there was some evidence of an additive quadratic effect of mean age of harem stallions on HSMDI and that DSI was operating externally to positively affect annual maximum harem size. Our results reveal the importance of intrinsic population processes as determinants of harem size in wildlife populations introduced and confined to large conservation reserves even under non‐stationary climatic conditions.

Recent studies have reported tight associations between social behaviour and ASR in various fish, bird and mammal species, including humans (Liker et al., 2013; Schacht et al., 2022), and the overwhelming support for ASR as a determinant of three of our phenology‐based harem size metrics was not surprising given the spatiotemporal dynamics of ASR and its importance in sexual selection and mating behaviour in polygynous mating systems (Clutton‐Brock & McAuliffe, 2009; Manning et al., 2015; Manning & McLoughlin, 2017; Székely et al., 2014). Previous studies on other ungulate species show that male‐skewed ASR can decrease the size and stability of breeding groups (Bender, 1996; Bonenfant et al., 2004; Kaseda & Khalil, 1996; L'Italien et al., 2012). The underlying mechanism of this negative relationship could be an increasing energetic demand for breeding males that acquire and maintain increasingly large harems in the presence of higher number of competitors (Clutton‐Brock et al., 1982; Roed et al., 2002). In our population, both annual mean harem size and annual maximum harem size decreased in response to increasing proportions of males. Additionally, the age of stallions when they first acquire a harem increased with increasing population size (K. Szemán, V. Kerekes, K. Brabender, & Zs. Végvári, unpublished data), indicating increased competition among males. High levels of male competition can also facilitate a decreased age in males that lose harems to stronger males, thus giving rise to a ∩‐shaped correlation between age, condition, resource holding abilities, dominance rank and paternity success (Mainguy & Côtè, 2008; Mysterud et al., 2003; Perlman et al., 2016; Setchell et al., 2006; Silk et al., 2020; Van Noordwijk & Van Schaik, 1985; Watts, 2019; Yoccoz et al., 2002). Thus, we posit that the negative effects of increasing male biased ASR on different phenology‐based harem size metrics may stem from the spatial and temporal dynamics of ASRs within a population, with underpinnings in population density, male physiology, behaviour, and age structure.

Our phenology‐based harem size metrics enabled us to elucidate the differential effects of mortality on various aspects of the 12‐month phenological harem size cycle. While cause‐specific mortality of adults was not a focus of this study, we did determine that adult mortality was largely attributed to disease, injury, or complications while giving birth. Higher adult mortality increased annual mean harem size. This was an unexpected pattern; we had predicted that increased adult mortality would decrease the number of mares in harems. However, we found that mean annual harem size was positively related to adult mortality. A plausible explanation for this is that annual births consistently exceeded annual adult mortalities during this period of positive population growth, producing net annual increases in mares as they matured and entered into the breeding age class in subsequent years, becoming members of existing and newly established harems each year despite increased mortalities as the population grew. Additionally, annual adult mortality was male biased (K. Szemán, V. Kerekes, K. Brabender, & Zs. Végvári, unpublished data), as is the case with many ungulates (Clutton‐Brock, 2016; Loison et al., 1999; Toïgo & Gaillard, 2003).

The negative effect of annual mean number of harems on dispersion of harem sizes coincided with the observed increase in population size and male abundance. As the population grew and number of males increased, so did male competition, leading to expectedly higher levels of mare harassment within harems and higher percentage of harem take‐overs (Ransom & Kaczensky, 2016). Such harassment by the harem holding stallions or bachelors can negatively impact mare fitness while harem take‐overs can negatively affect within‐harem social cohesion, which has been shown to reduce the reproductive performance of mares (Cameron et al., 2009; Linklater et al., 1999; Sunderesan et al., 2007). Potentially, these conditions can induce a higher rate of secondary dispersal by mares, which may lead to increasing numbers of consistently smaller harems.

We used HSMDI as an index to describe the number of months mean harem size in a given year was below the 15‐year average. In our population, the 15‐year average harem size was 3.46 adult mares per harem. Harem size was above the 15‐year average during the first years of the study, then it began to decrease, eventually falling below the average in 2002 and staying at this level until the end of the study period, except one peak in 2012. We found that ASR had a major negative impact on HSMDI and some evidence that mean age of harem stallions may also have played a measurable role. We hypothesise that these associations were driven by population trends. Despite a steady increase in population size observed during the study period (Kerekes et al., 2021) that resulted in an increased proportion of males, the ASR remained female biased. Mean harem size was expected to increase in parallel with population size. Instead, the number of harems increased, revealing a threshold in harem size. Interestingly, we would have overlooked these demographic factors and patterns if we had only considered the static view of harem size (e.g. used only the annual mean harem size for this analyses).

The positive effect of drought severity on maximum harem size detected in our competing model was in line with expectations given demonstrated impacts of climate and resources on polygyny thresholds in feral horses (Manning & McLoughlin, 2017). We propose two mutually non‐exclusive hypotheses that may explain why this effect was not stronger. First, the Pentezug Reserve is relatively large, providing Przewalski horses the opportunity to spatially shift to track resources and select favourable weather conditions, thereby diminishing the extrinsic influence of drought. Moreover, the tallgrass steppe system supports year‐round environmental conditions that are suitable for horses, which may reduce the need for long‐distance migration while maintaining the evolutionarily adapted social organisation and group size (Szemán et al., 2021). Second, foraging and water resources were available and abundant year‐round in the study area, and the body condition of horses did not change over the years, possibly dampening the underlying link between annual weather conditions and water forage resources (e.g. habitat quality) among males and mate selection by females (Brabender et al., 2016).

In conclusion, our study deepens our knowledge of harem size phenology in herbivores living in harems. It provides a unique opportunity to simultaneously investigate how demographic parameters of a population and environmental factors shape harem size and the different ways in which we may measure this across its 12‐month phenological cycle. Our results raise interesting questions regarding the social structure of a semi‐wild population of a polygynous herbivore. Although further work on harem phenology across species living in year‐round harems in a more unpredictable environment and effect of predation on harem phenology will provide additional insights into selection beyond our current state of knowledge.

## AUTHOR CONTRIBUTIONS

**Karola Szemán:** Conceptualization (equal); data curation (lead); formal analysis (lead); methodology (equal); visualization (equal); writing – original draft (lead); writing – review and editing (equal). **Zsolt Végvári:** Conceptualization (equal); data curation (supporting); formal analysis (supporting); methodology (equal); supervision (equal); visualization (equal); writing – review and editing (equal). **Szilvia Gőri:** Conceptualization (equal); writing – review and editing (equal). **István Kapocsi:** Conceptualization (equal); writing – review and editing (equal). **Tamás Székely:** Conceptualization (equal); methodology (equal); supervision (equal); writing – review and editing (equal). **Jeffrey A. Manning:** Conceptualization (equal); data curation (equal); formal analysis (equal); methodology (lead); visualization (equal); writing – original draft (supporting); writing – review and editing (lead).

## CONFLICT OF INTEREST STATEMENT

The authors declare that they have not known competing interest that could have appeared to influence the work reported in this manuscript.

## Supporting information


Appendix S1.

## Data Availability

All relevant data are within the paper and its Supporting Information section.
