# Supplementary material for: Harem size should be measured by more than the sum of its parts: Phenology‐based measurements reveal joint effects of intrinsic and extrinsic factors on a polygamous herbivore under non‐stationary climatic conditions
Source: Ecol Evol. 2024 Feb 5;14(2):e10865. doi: 10.1002/ece3.10865 (PMC10844713; doi:10.1002/ece3.10865)

Supplementary Material

<u>Table S1:</u> Results of Spearman correlation test among predictors, using a correlation threshold of r>0.7 to avoid collinearity in linear regression models (Dormann *et al*., 2013). There was no evidence of correlation among variables.

	Annual DSI	Annual mean number of harems	ASR	Mean age of harem stallions	Annual adult mortality	Annual juvenile female mortality
Annual DSI		0.21	-0.11	-0.1	0.47	-0.03
Annual mean number of harems	0.21		0.55	-0.3	-0.62	0.25
ASR	-0.11	0.55		-0.57	-0.64	0.42
Mean age of harem stallions	-0.1	-0.3	-0.57		0.39	0.03
Annual adult mortality	0.47	-0.62	-0.64	0.39		-0.13
Annual juvenile female mortality	-0.03	0.25	0.42	0.03	-0.13	

Figure S1: Tendency of harem size variation over the study period for a naturally regulated population of Przewalski horses in Hortobágy National Park, Hungary.

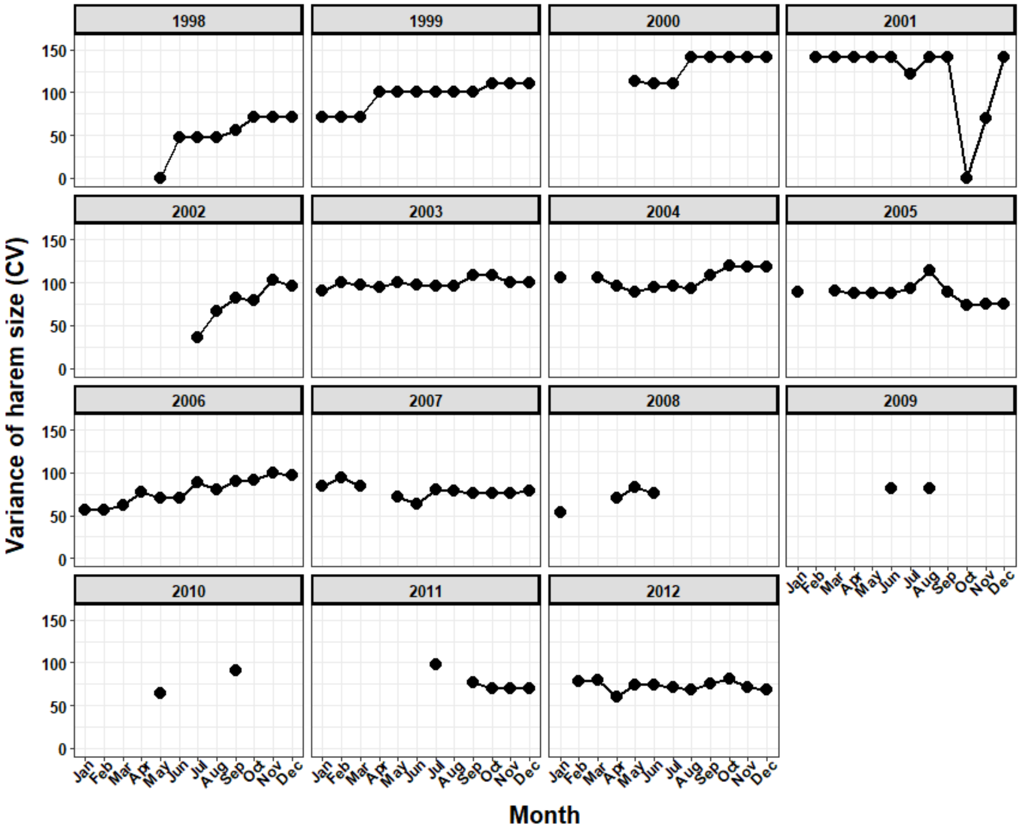

Figure S2: Diagnostic plots of the top model containing annual mean harem size as response variable. The predictors of the model were adult sex ratio (ASR) and annual adult mortality.

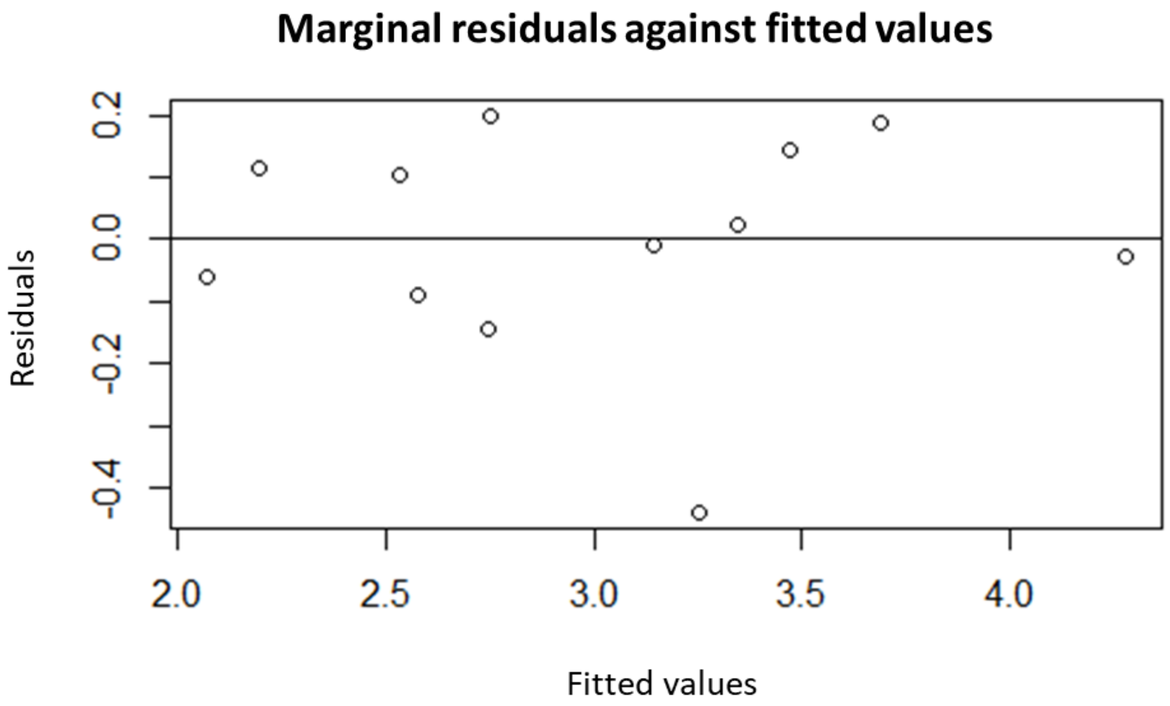

Figure S3: Diagnostic plots of the second best model containing annual maximum harem size as response variable. The predictor of the model was adult sex ratio (ASR).

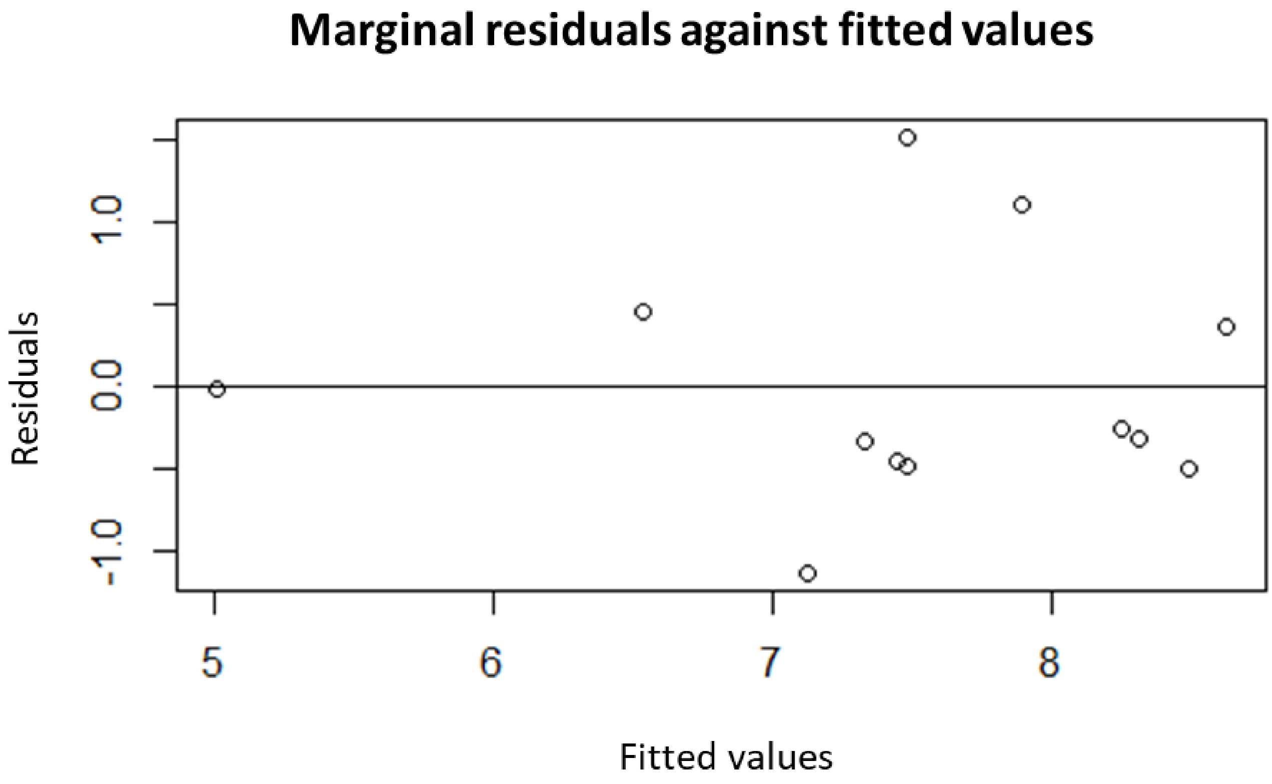

Figure S4: Diagnostic plots of the top model contained variation of harem size as response variable. The predictor of the model was annual mean number of harems.

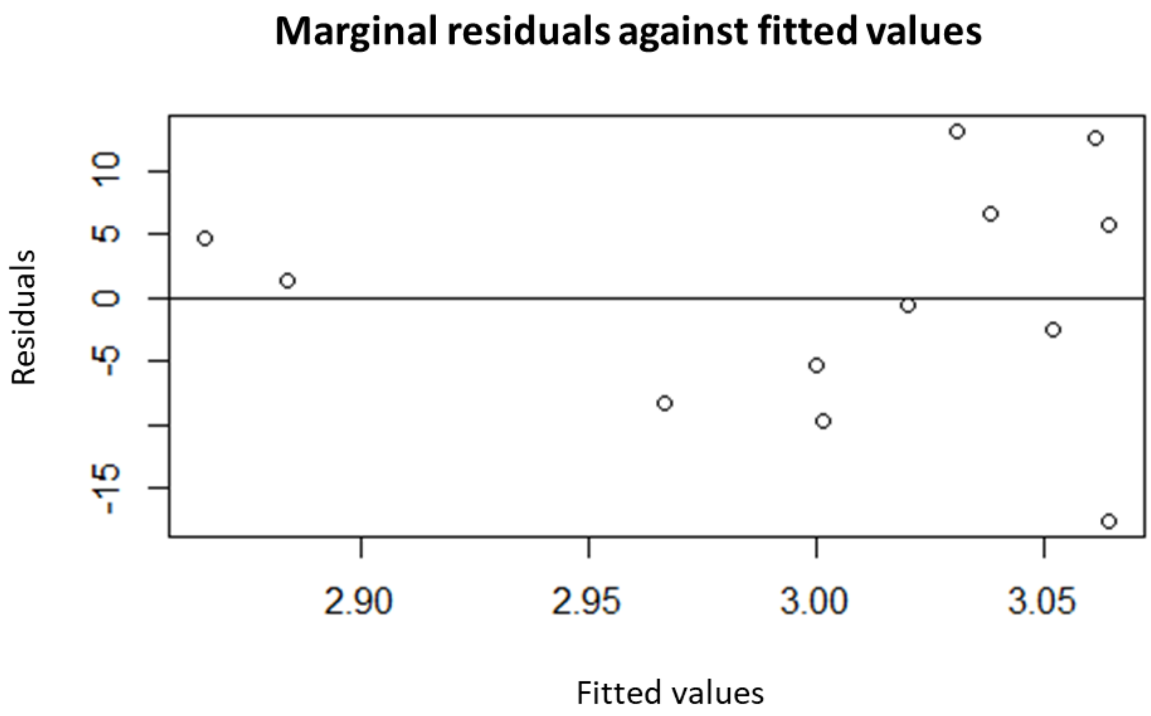

Figure S5: Diagnostic plots of the top model contained harem size monthly departure index (HSMDI) as response variable. The predictor of the model was adult sex ratio (ASR).

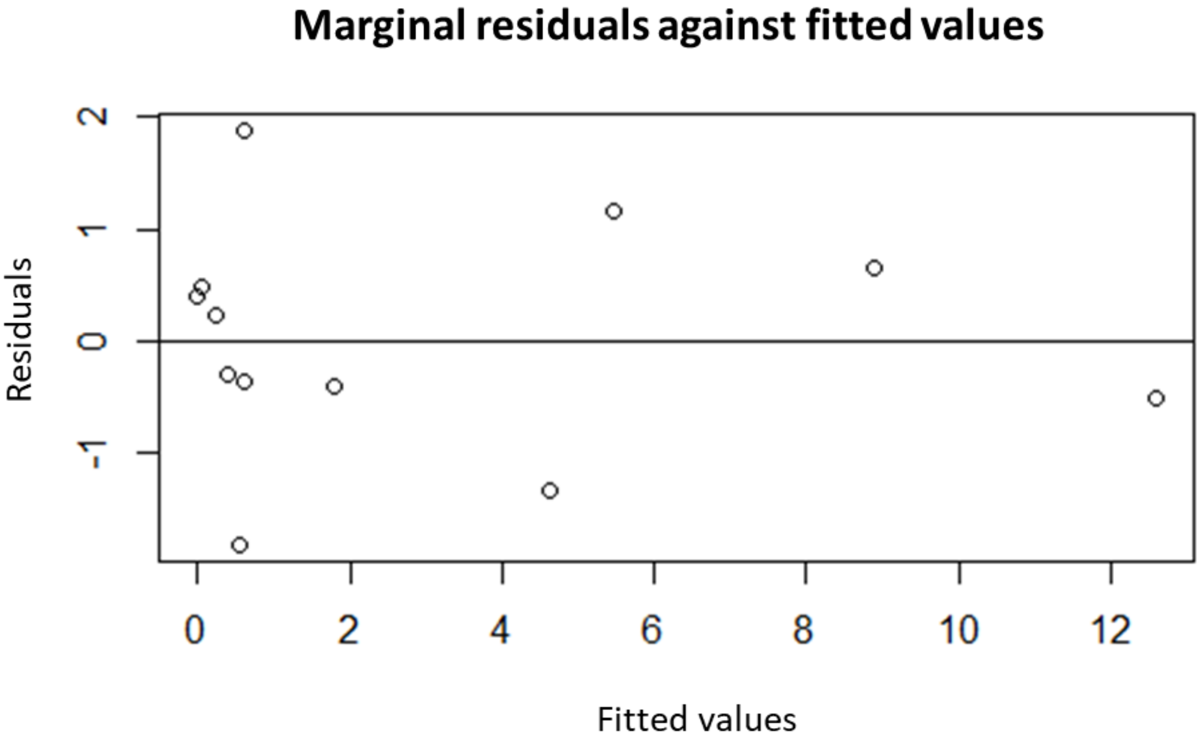

Figure S6: Two-dimensional relationships between annual mean harem size and two covariates occurring in a 3-dimensional additive ordinary least squares regression model (ΔAICc = 0.0; Table 3, Figure 4). Shaded area is 95% CI.

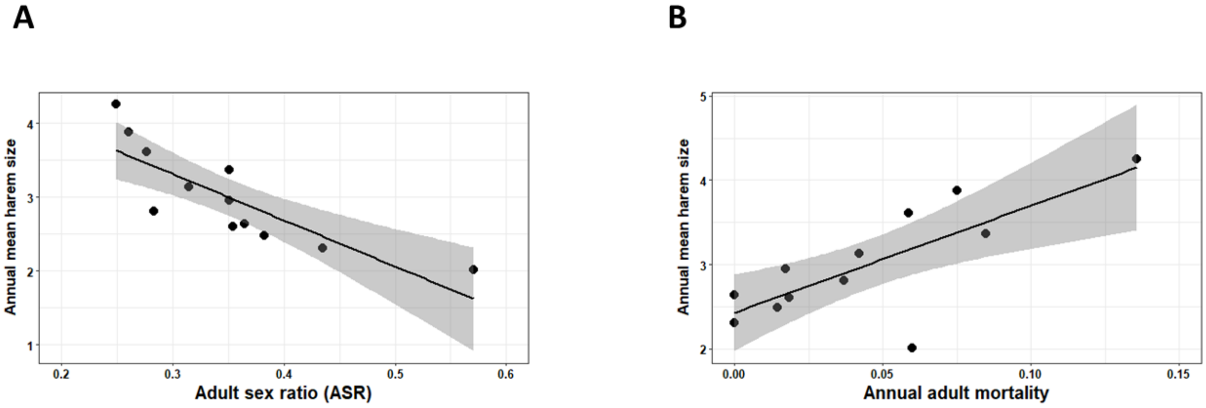

Figure S7: Two-dimensional relationships between annual maximum harem size and three covariates occurring in three competing ordinary least squares regression models (ΔAICc <2.0; Table 3, Figure 5). Shaded area is 95% CI.

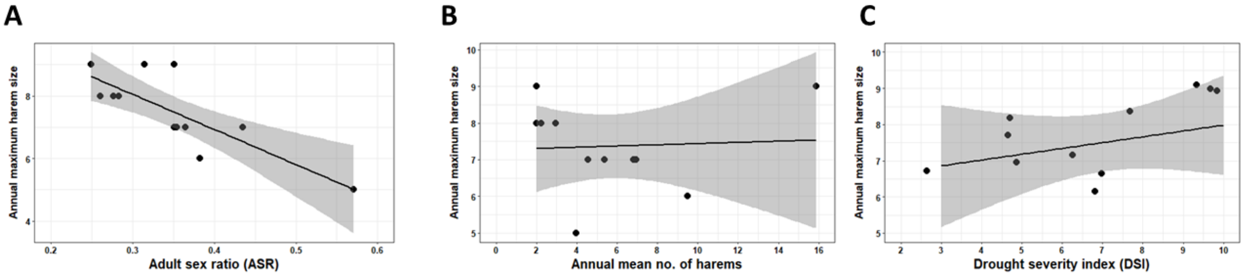

Supplement: Supplementary file 1 — Appendix S1. [file ECE3-14-e10865-s001.pdf]
